# Oxidative stress differentially impacts apical and basolateral secretion of angiogenic factors from human iPSC-derived retinal pigment epithelium cells

**DOI:** 10.1038/s41598-022-16701-6

**Published:** 2022-07-26

**Authors:** Lisheng Chen, N. Dayanthi Perera, Athanasios J. Karoukis, Kecia L. Feathers, Robin R. Ali, Debra A. Thompson, Abigail T. Fahim

**Affiliations:** 1 Department of Ophthalmology and Visual Sciences, University of Michigan, Ann Arbor, MI 48105 USA; 2 KCL Centre for Cell and Gene Therapy, London, WC2R 2LS England UK; 3 Department of Biological Chemistry, University of Michigan, Ann Arbor, MI 48105 USA

**Keywords:** Retinal diseases, Cell polarity, Mechanisms of disease, Extracellular signalling molecules

## Abstract

The retinal pigment epithelium (RPE) is a polarized monolayer that secretes growth factors and cytokines towards the retina apically and the choroid basolaterally. Numerous RPE secreted proteins have been linked to the pathogenesis of age-related macular degeneration (AMD). The purpose of this study was to determine the differential apical and basolateral secretome of RPE cells, and the effects of oxidative stress on directional secretion of proteins linked to AMD and angiogenesis. Tandem mass tag spectrometry was used to profile proteins in human iPSC-RPE apical and basolateral conditioned media. Changes in secretion after oxidative stress induced by H_2_O_2_ or tert-butyl hydroperoxide (tBH) were investigated by ELISA and western analysis. Out of 926 differentially secreted proteins, 890 (96%) were more apical. Oxidative stress altered the secretion of multiple factors implicated in AMD and neovascularization and promoted a pro-angiogenic microenvironment by increasing the secretion of pro-angiogenic molecules (VEGF, PTN, and CRYAB) and decreasing the secretion of anti-angiogenic molecules (PEDF and CFH). Apical secretion was impacted more than basolateral for PEDF, CRYAB and CFH, while basolateral secretion was impacted more for VEGF, which may have implications for choroidal neovascularization. This study lays a foundation for investigations of dysfunctional RPE polarized protein secretion in AMD and other chorioretinal degenerative disorders.

## Introduction

Despite advances in treatments, AMD remains the leading cause of blindness in the United States in adults over the age of 65^1^. Although the primary pathomechanisms of AMD are debated, the disease is characterized at the cellular level by RPE dysfunction and metabolic stress, accumulation of sub-RPE deposits, overlying photoreceptor death, and underlying choroidal endothelial cell death and subsequent choroidal thinning. The retinal pigment epithelium (RPE) is a prolific secretor of growth factors and cytokines in a polarized manner towards the photoreceptors on the apical side and the choroidal vasculature on the basolateral side. Disturbances in RPE directional protein secretion have been implicated in chorioretinal degeneration, including AMD^2^. These prior studies show that RPE cells secrete vascular endothelial growth factor (VEGF), which promotes angiogenesis, toward the choroid, and pigment epithelium derived factor (PEDF), which inhibits angiogenesis, toward the photoreceptors^3^. Additional proteins with AMD risk alleles, such as complement factor H and fibulin 5, are also directionally secreted from RPE cells^2,4^. Furthermore, the RPE demonstrates directional secretion of proteins encoded by Mendelian disease genes, such as *TIMP3* and *EFEMP1*, which cause Sorsby macular dystrophy and Doyne honeycomb macular dystrophy, respectively^5,6^.

The polarized secretome of RPE cells has not been fully documented, and it remains unknown what additional RPE secreted proteins may impact retinal and choroidal health. Many previous studies of the RPE secretome have used conventional cell culture on plastic wells and thus have investigated only apical conditioned media and have not captured basolateral secreted proteins, which may impact both choroidal neovascularization and choroidal degeneration in geographic atrophy^7–9^. More recent studies have investigated both apical and basolateral protein secretion, since understanding the differential polarized secretome of RPE cells is foundational to investigations of dysfunctional protein secretion in AMD and other chorioretinal degenerative diseases^10,11^. The purpose of this study is to further investigate the directional secretion of RPE proteins, and how oxidative stress may impact polarized secretion of proteins important in AMD. A human induced pluripotent stem cell (iPSC)-derived RPE line was grown on permeable supports, allowing assessment of both the apical and basolateral profile of secreted proteins, focusing specifically on those proteins known to function extracellularly and therefore potentially impacting the adjacent photoreceptors and choroidal vasculature. Using H_2_O_2_ and tert-butyl hydroperoxide (tBH) oxidative stress models, we demonstrate changes in the directional secretion of RPE proteins that could contribute to neovascularization.

## Results

### RPE directional protein secretion is primarily apical

**Figure 1:**
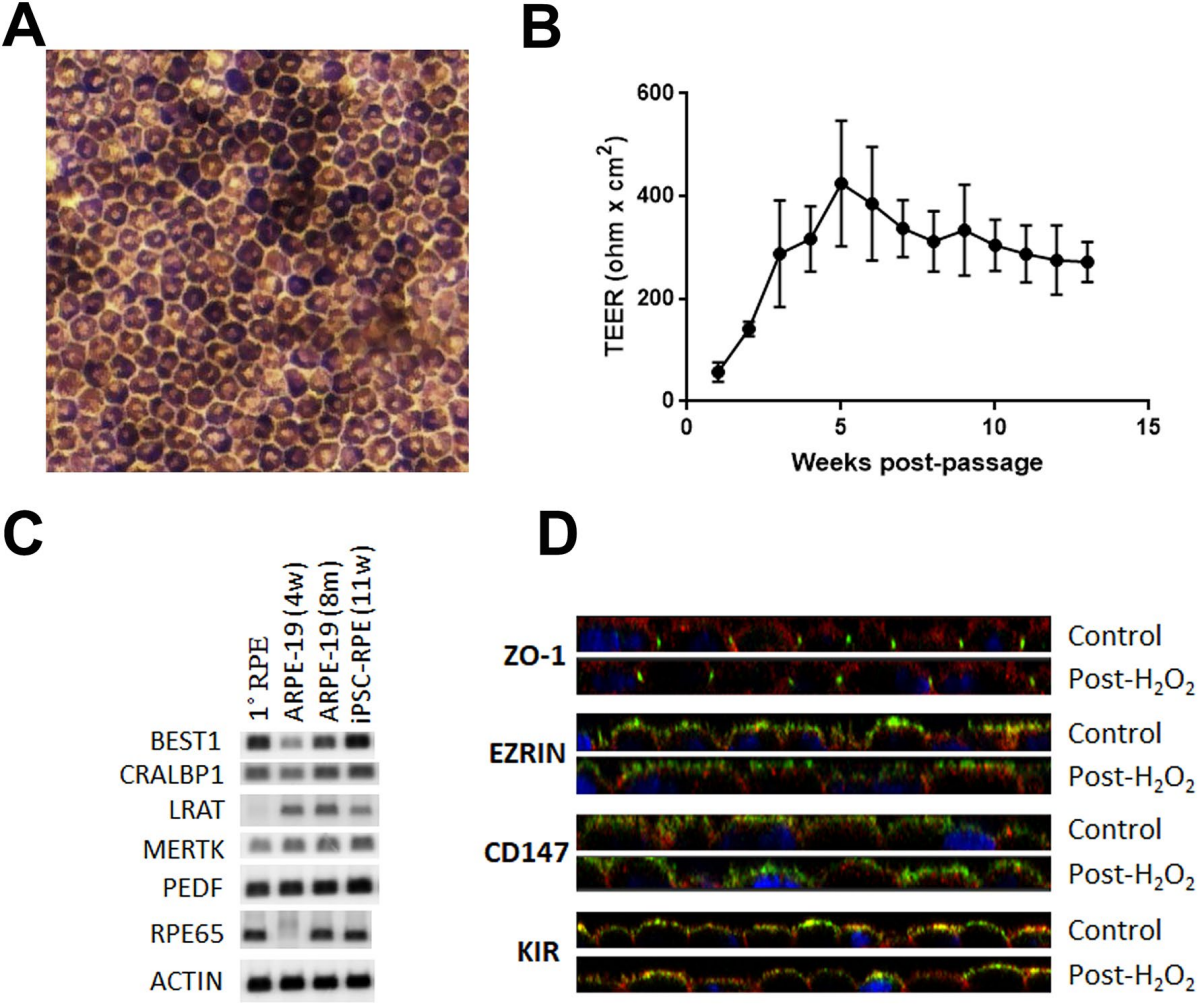
Human iPSC-RPE demonstrates appropriate polarity and differentiation, (**A**) with melanin pigmentation and cobblestone morphology, (**B**) and rise in TEER after passage onto Transwell filters. N = 6–65 biologic replicates depending on the time point, no technical replicates. Data points show mean ± SD. (**C**) RT-PCR of RPE markers in iPSC-RPE 11 weeks post-passage compared to primary adult human RPE and ARPE19 cells at 4 weeks and 8 months post-passage (uncropped images are shown in Supplementary Fig. 3). (**D**) ICC of iPSC-RPE for ZO-1 at tight junctions, and CD147, Ezrin, and Kir7.1 in the apical membrane. Phalloidin is red and DAPI is blue. Staining is shown both before (control) and after (Post-H_2_O_2_) exposure to 800 µM H_2_O_2_ for 24 h.

iPSC-derived RPE cells (iPSC-RPE) were maintained on permeable supports and used between 2 and 6 months post-passage. iPSC-RPE had appropriate melanin pigmentation and cobblestone morphology and rise in TEER starting at 2 weeks post-passage, with plateau at an average of 312 Ω cm^2^ by 8 weeks (Fig. 1A,B). Cells showed expression of RPE markers and appropriate localization of membrane proteins (Fig. 1C,D). iPSC-RPE cells were maintained in serum-free media containing B27 supplement with albumin. To analyze the secretome using mass spectrometry, without large amounts of albumin confounding the results, a B18 supplement was formulated similar to B27 without albumin. iPSC-RPE in B18-supplemented media overnight maintained TEER similar to standard B27-supplemented media (Supplementary Fig. 1).

**Figure 2:**
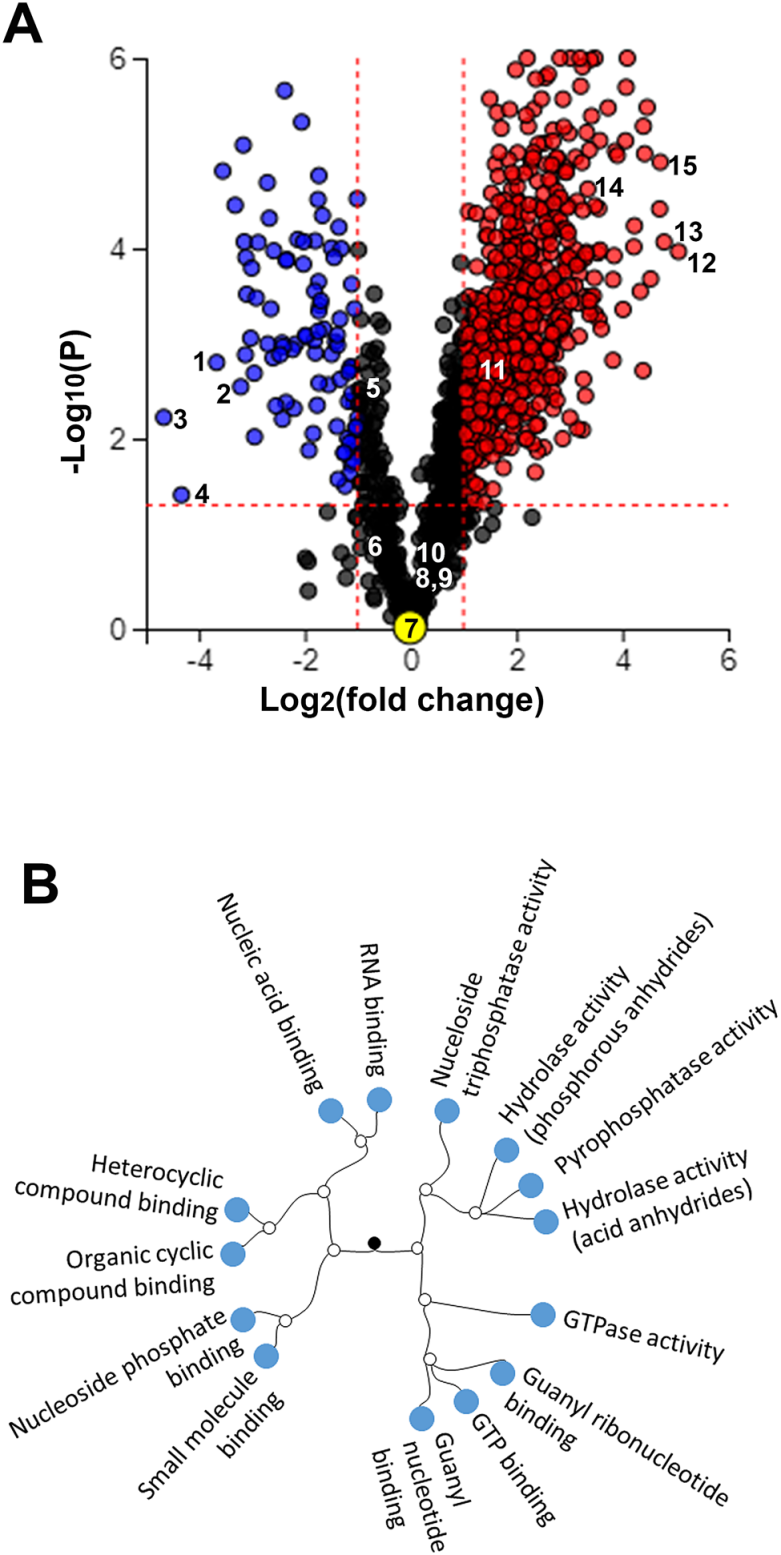
Human iPSC-RPE polarized secretome is more apical than basolateral. (**A**) Volcano plot from data set 2 showing proteins with at least twofold change and p < 0.05, in blue (more basolateral) or red (more apical). N = 4 biologic replicates. The 3 most differentially secreted proteins in both the apical and basolateral compartments, as well as proteins of interest as described in the results section, are labeled by number on the graph: 1. RNF7, 2. TIMP3, 3. UBR, 4. PTPN22, 5. CFI, 6. VEGF, 7. C1QTNF5, 8. PTN, 9. HTRA1, 10. CFH, 11. PEDF, 12. RPL12, 13. HMGN2, 14. CRYAB, 15. PSMA1. (**B**) Dendrogram from data set 2 showing molecular functions that were differentially represented in apical vs basolateral samples (p < 0.05, adjusted for false discovery rate). Analysis was performed using Advaita Bio ipathwayguide^12,13^.

Conditioned B18 media was collected and concentrated after 16 h from apical and basolateral chambers of triplicate sets of wells and tandem mass tag spectrometry was performed. The experimental design is illustrated in Supplementary Fig. 2. To account for the difference in volume between apical and basolateral chambers, the raw counts from mass spectrometry were adjusted according to concentration factor, in order to compare the total amount of each protein secreted apically vs basolaterally. A total of 1421 secreted proteins were identified. Using criteria of at least twofold difference and a p-value < 0.05 (adjusted for false discovery rate), 926 proteins had differential secretion between the apical and basolateral compartments. For reproducibility, a second data set was obtained using 4 replicate sets of wells, using equal volumes of media (800 mL) in both apical and basolateral chambers. The second data set showed overall congruence with the first. A total of 1320 secreted proteins were identified (compared to 1421), and 732 proteins showed differential directional secretion (compared to 926). The lower number of proteins is due to missing values, which is more likely with increasing number of samples. Overall, 87% (637/732) of differentially secreted proteins in data set 2 were secreted more apically (Fig. 2A). The average number of unique peptides per protein was 4.2 in data set 1 vs 4.8 in data set 2 (p < 0.01). Given the higher number of replicates and higher number of unique peptides detected per protein, data set 2 was used for Tables 1, 2, 3. Pathway analysis using Advaita iPathwayGuide demonstrated that molecular functions involving nucleic acid and nucleic acid substrate binding, as well as GTPase activity were significantly enriched in the differentially secreted proteins (Fig. 2B)^12,13^. G proteins and GTPase activity are important in numerous cell processes, including the visual cycle and membrane trafficking, and nucleic acid sensing proteins in RPE cells may be important in inflammation and age-related macular degeneration^14^.

**Table 1:** Directional secretion of RPE effector proteins.

Category	Protein	A/BL
Growth factors and cytokines	LGALS1 (galectin 1)	10.0***
	MIF (macrophage migration inhibitory factor)	7.7****
	HDGF (heparin binding growth factor)	5.3*****
	ANXA2 (annexin A2)	5.3****
	GPI (glucose-6-phosphate isomerase)	4.9****
	ECM1 (extracellular matrix protein 1)	4.4***
	IGFALS (insulin like growth factor binding protein acid labile subunit)	3.4***
	ERBB4 (erb-b2 receptor tyrosine kinase 4)	3.2*
	MFGE8 (milk fat globule EGF and factor V/VIII domain containing)	3.1***
	FAM3C (FAM3 metabolism regulating signaling molecule C)	2.4**
	**IGFBP5 (insulin like growth factor binding protein 5)**	0.1****
Proteases	PSMA1 (proteasome 20S subunit alpha 1)	26.3****
	PSMA5 (proteasome 20S subunit alpha 5)	17.1*****
	PSMA2 (proteasome 20S subunit alpha 2)	14.6*****
	PSMA4 (proteasome 20S subunit alpha 4)	8.1*****
	PEPD (peptidase D)	6.6****
	LTA4H (leukotriene A4 hydrolase)	6.4***
	CPQ (carboxypeptidase Q)	6.0****
	PDIA3 (protein disulfide isomerase family A member 3)	5.8***
	PRCP (prolylcarboxypeptidase)	5.2****
	CNDP2 (carnosine dipeptidase 2)	4.8**
	AGA (aspartylglucosaminidase)	4.7**
	PSMD2 (proteasome 26S subunit, non-ATPase 2)	4.3****
	PSMA7 (proteasome 20S subunit alpha 7)	4.0*****
	PSMA6 (proteasome 20S subunit alpha 6)	3.8****
	DPP7 (dipeptidyl peptidase 7)	3.6****
	PSMD7 (proteasome 26S subunit, non-ATPase 7)	3.5**
	CTSV (cathepsin V)	3.4**
	PSMB6 (proteasome 20S subunit beta 6)	3.4****
	PSMD1 (proteasome 26S subunit, non-ATPase 1)	3.4**
	PSMB3 (proteasome 20S subunit beta 3)	3.3*****
	CTSC (cathepsin C)	3.2**
	GGH (gamma-glutamyl hydrolase)	3.2***
	PSMB7 (proteasome 20S subunit beta 7)	3.2****
	PSMB5 (proteasome 20S subunit beta 5)	3.2****
	**CTSO (cathepsin O)**	0.2**
Protease inhibitors	CRYAB (crystallin alpha B)	10.2****
	SERPINB9 (serpin family B member 9)	6.7**
	CSTB (cystatin B)	5.7*****
	SERPINE3 (serpin family E member 3)	4.2**
	SERPINF2 (serpin family F member 2)	3.2**
	SERPINE1 (serpin family E member 1)	3.0**
	**SERPINC1 (serpin family C member 1)**	0.2***
	**TIMP3 (TIMP metallopeptidase inhibitor 3)**	0.1**
Extracellular matrix	COL14A1 (collagen type XIV alpha 1 chain)	5.1**
	COL6A2 (collagen type VI alpha 2 chain)	4.2***
	COL6A1 (collagen type VI alpha 1 chain)	3.8***
	CHI3L1 (chitinase 3 like 1)	3.5**
	COL11A1 (collagen type XI alpha 1 chain)	3.3**
	TGFBI (transforming growth factor beta induced)	3.2*
	**COL8A1 (collagen type VIII alpha 1 chain)**	0.3***
Complement cascade	CD81 (CD81 molecule)	6.3**
	HSP90AB1 (heat shock protein 90 alpha family class B member 1)	4.8***
Lipid homeostasis	FABP3 (fatty acid binding protein 3)	4.6***
	ANGPTL4 (angiopoietin like 4)	3.5****
	**APOC3 (apolipoprotein C3)**	0.2***
	**PCSK9 (proprotein convertase subtilisin/kexin type 9)**	0.2**

A/BL = apical to basolateral ratio; proteins are listed from highest to lowest A/BL within each category. Due to size constraints, only proteins with at least threefold difference between apical and basolateral are shown. Bold highlights proteins with more basolateral than apical secretion. P-values were calculated with a two-tailed student’s T-test with a Benjamin-Hochberg adjustment. P-value < 0.05 (*), < 0.01 (**), < 0.001 (***), < 0.0001 (****), or < 0.00001 (*****).

**Table 2:** RPE directional secretion of proteins encoded by disease genes.

Disease	Protein	A/BL
**Mendelian posterior segment disease**		
Oculocutaneous albinism (OCA)	TYRP1	3.7**
Tay-Sachs	HEXA	3.4***
Stickler syndrome	COL11A1	3.3**
Stickler syndrome	COL9A2	2.4**
Cone-rod dystrophy	CDHR1	2.0**
Retinitis pigmentosa (RP)	SNRNP200	2.0**
Late onset retinal degeneration (LORD)	C1QTNF5	1.0
Doyne honeycomb macular dystrophy	**EFEMP1**	**0.9**
Cone-rod dystrophy	**ADAM9**	0.5**
Microphthalmia; retinal dystrophy, iris coloboma, and comedogenic acne syndrome	**RBP4**	0.4***
Sorsby macular dystrophy	**TIMP3**	0.1**
**Mendelian anterior segment disease**		
Congenital cataract	CRYAB	10.2****
Mucopolysaccharidosis type IV (with corneal clouding)	GLB1	7.5***
Primary open angle glaucoma (POAG)	OPTN	5.3**
Congenital Cataract	VIM	4.4***
Corneal dystrophies	TGFBI	3.2*
Amyloidosis, meretoja syndrome; lattice corneal dystrophy	GSN	2.0*
Gaucher disease (with corneal opacities)	GBA	2.0*
**Other Mendelian ophthalmic disease**		
Microphthalmia	ALDH1A3	5.4***
Progressive external ophthalmoplegia	RRM2B	4.8***
Age-related macular degeneration risk loci	C3	1.5*
	FBLN5	1.3
	CFH	1.3
	HTRA1	1.2
	**CFI**	0.6**
	**TIMP3**	0.1**

A/BL = apical to basolateral ratio; proteins are listed from highest to lowest A/BL within each category. Bold highlights proteins with more basolateral than apical secretion. P-values were calculated with a two-tailed student’s T-test with a Benjamin-Hochberg adjustment. P-value < 0.05 (*), < 0.01 (**), < 0.001 (***), < 0.0001 (****), or > 0.05 (non-significant, no asterisk).

**Table 3:** RPE directional secretion of angiogenic factors.

Protein	A/BL
**Positive regulation**	
CRYAB (crystallin alpha B)	10.2****
ANXA3 (annexin A3)	7.7***
ECM1 (extracellular matrix protein 1)	4.4***
ANGPTL4 (angiopoietin like 4)	3.5****
ANXA1 (annexin A1)	3.5***
SERPINE1 (serpin family E member 1)	3.0**
GRN (granulin precursor)	2.8***
MYDGF (myeloid derived growth factor)	2.7**
SFRP1 (secreted frizzled related protein 1)	0.8
VEGFA (vascular endothelial growth factor A)	0.7
**CX3CL1 (C-X3-C motif chemokine ligand 1)**	0.4*
**Negative regulation**	
PGK1 (phosphoglycerate kinase 1)	7.1*****
TGFBI (transforming growth factor beta induced)	3.2*
SERPINF1 (serpin family F member 1; aka PEDF)	2.9**
COL4A2 (collagen type IV alpha 2 chain)	2.5***
NAXE (NAD(P)HX epimerase)	2.1**
FN1 (fibronectin 1)	1.8**
THBS1 (thrombospondin 1)	1.4
PTN (pleiotrophin)	1.2
TGFB2 (transforming growth factor beta 2)	0.8
**THBS4 (thrombospondin 4)**	0.6*
**SULF1 (sulfatase 1)**	0.5**
**RNH1 (ribonuclease/angiogenin inhibitor 1)**	0.2**

A/BL = apical to basolateral ratio; proteins are listed from highest to lowest A/BL within each category. Bold highlights proteins with more basolateral than apical secretion. P-values were calculated with a two-tailed student’s T-test with a Benjamin-Hochberg adjustment. P-value < 0.05 (*), < 0.01 (**), < 0.001 (***), < 0.0001 (****), or > 0.05 (non-significant, no asterisk).

To consider the potential effects of RPE secreted proteins on the adjacent tissues (the neurosensory retina and the choroidal vasculature), attention was focused on “effector proteins,” those that are known to function extracellularly and non-autonomously, thereby excluding primarily intracellular proteins that are secreted as cellular waste or surplus. We included the categories of growth factors and cytokines, proteases, protease inhibitors, extracellular matrix components, complement cascade proteins, and lipid homeostasis proteins. Annotation in Advaita was used to identify proteins in each category and was cross-referenced with UniProt to identify extracellular proteins (Table 1)^12,15^. The largest category of protein was proteases. Similar to the overall trend of the polarized secretome seen in Fig. 2A, the overwhelming majority of these proteins were secreted apically more than basolaterally.

### Disease-associated proteins are directionally secreted

Several known disease genes were secreted by RPE cells, shown in Table 2. Mendelian disease genes that were differentially secreted were identified by annotation in Advaita, and additional AMD risk loci are included in Table 2 as well. An important protease inhibitor, TIMP3, was secreted tenfold more into the basolateral chamber (p < 0.01). Mutations in *TIMP3* cause Sorsby’s macular dystrophy, an autosomal dominant macular dystrophy with early onset macular atrophy and severe aggressive choroidal neovascularization. Risk alleles for AMD have also been identified in *TIMP3*^16^. Late onset retinal degeneration (LORD), a disease with chorioretinal degeneration of the posterior pole in late adulthood, is caused by mutations in *C1QTNF5*, encoding a protein of unknown function. C1QTNF5 protein was secreted equally in apical and basolateral directions by RPE cells. *CFH* and *HTRA1/ARMS2* are the most well-established risk loci for AMD^17–19^. CFH And HTRA1 proteins were both secreted from RPE, similarly in apical and basolateral directions. CFI which also has AMD risk alleles, was secreted more basolateral than apical (p < 0.01)^18^. CFH has been shown to inhibit neovascularization, and loss of function of CFH is associated with increased complement activation, inflammation, and increased risk of AMD^18–20^. HTRA1 is a serine protease and is known to degrade EFEMP1 and THBS1, both secreted from RPE cells^21^.

Other proteins important for the regulation of angiogenesis were also directionally secreted (Table 3). VEGF showed a non-significant trend towards directional basolateral secretion. PEDF, which inhibits angiogenesis and induces endothelial cell apoptosis, was secreted 2.9-fold more apical than basolateral (p < 0.01). CRYAB, which inhibits caspase 3 and is neuroprotective to photoreceptors and also promotes VEGF signaling and angiogenesis, was directionally secreted by RPE 10.2-fold more apical than basolateral (p < 0.0001)^2^. PTN, which plays a complex role in angiogenesis in multiple diseases, including cancer, likely plays a pro-angiogenic role in diabetic retinopathy^22,23^. PTN was secreted similarly in apical and basolateral directions.

### Oxidative stress alters RPE directional secretion of proteins implicated in angiogenesis and AMD

**Figure 3:**
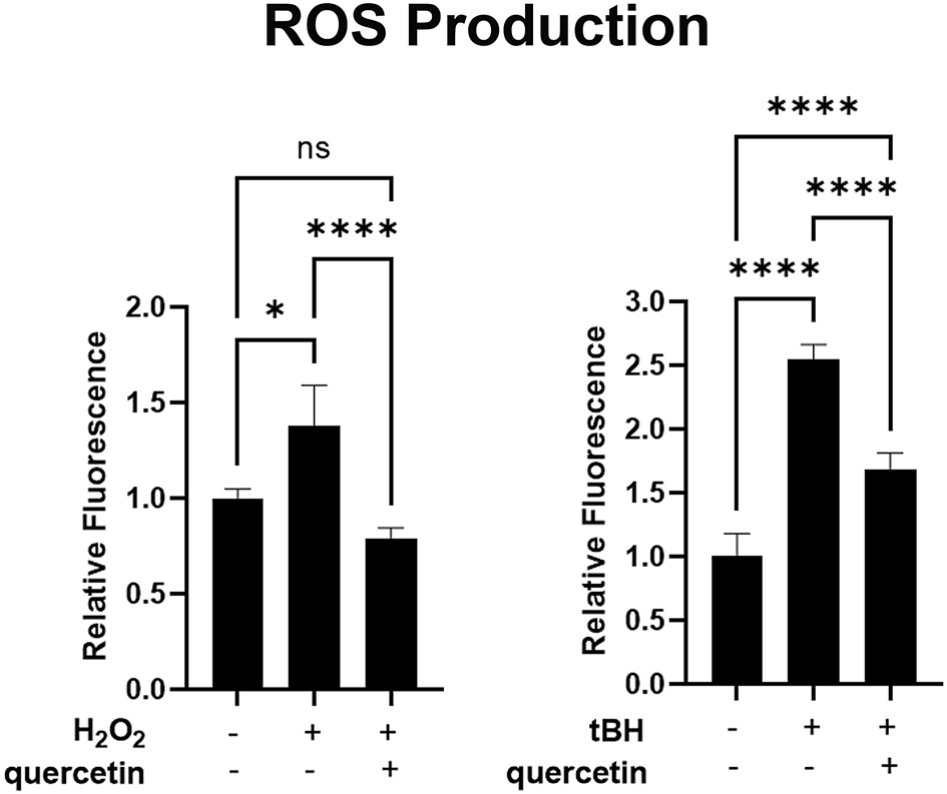
Reactive Oxygen Species (ROS) production after oxidative stress in iPSC-RPE. Graph shows relative fluorescence detection after iPSC-RPE are exposed to either 800 µM H_2_O_2_ or 1 mM tBH, with or without pre-treatment with 400 µM quercetin, using Carboxy-H_2_DCFDA as a fluorescent cell-permeant indicator of ROS. For the H_2_O_2_ experiments, n = 12 for the H_2_O_2_ group and n = 6 for the control group and the H_2_O_2_ + quercetin group. For the tBH experiments, n = 5 for all groups. All replicates were biologic; there were no technical replicates. Results were analyzed by Kruskal–Wallis for the H_2_O_2_ experiments due to unequal sample size, and by one-way ANOVA for the tBH experiments. *p < 0.05, ****p < 0.0001, ns = not significant.

To investigate the effects of oxidative stress on directional protein secretion, cells were exposed to H_2_O_2_ or tBH. H_2_O_2_ has been used extensively to model oxidative stress in AMD and was previously shown to increase RPE secretion of VEGF^24,25^. tBH was used as a second oxidative agent based on prior reports of superior reactive oxygen species (ROS) production in ARPE19 cells with tBH compared to H_2_O_2_^26^. iPSC-RPE cells were treated with 800 µM H_2_O_2_ for 24 h, or 1 mM tBH for 6 h, with or without pre-treatment with quercetin, an ROS scavenging agent which has been previously used to protect RPE cells from oxidative stress^27^. Reactive oxygen species (ROS) production was measured using carboxy-H_2_DCFDA, a cell permeable reagent that fluoresces after reaction with intracellular ROS (Fig. 3). iPSC-RPE ROS significantly increased after exposure to H_2_O_2_ or tBH, and the effect was rescued by ROS scavenging by quercetin in each case. The longer incubation of 24 h was chosen for H_2_O_2_, because no ROS were detected after a 6 h incubation (data not shown). TEER of iPSC-RPE recovered to baseline within 48 h after either treatment, indicating overall health and viability of the cells.

**Figure 4:**
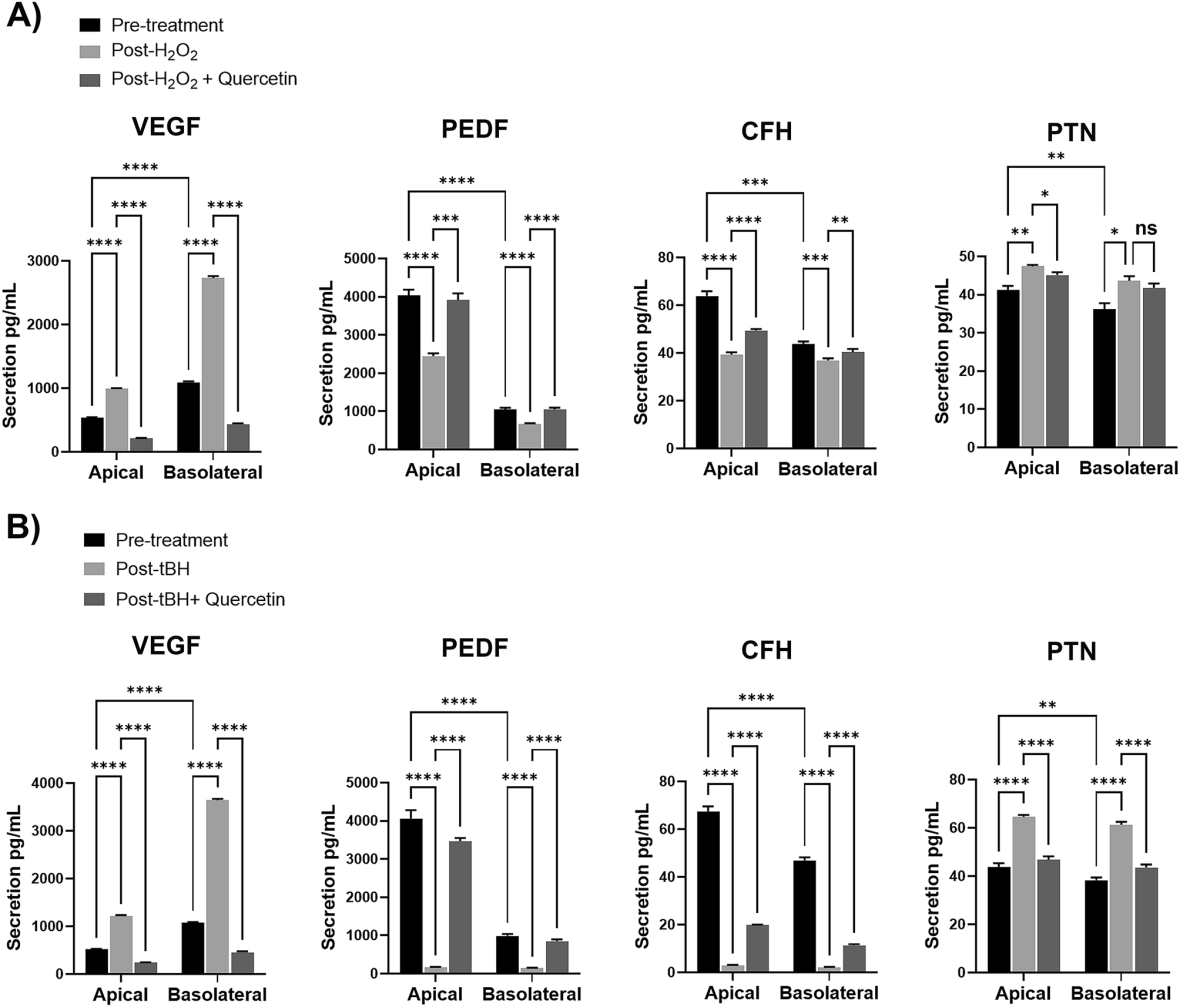
H_2_O_2_-induced oxidative stress alters RPE polarized secretion of angiogenic regulatory factors. Bar graphs show differences in apical and basolateral secretion of VEGF, PEDF, CFH, and PTN before (Pre-treatment, black) and after incubation with (**A**) 800 µM H_2_O_2_ for 24 h (Post-H_2_O_2_, light grey), or (**B**) 1 mM tBH for 6 h (Post-tBH, light grey). Dark grey bars show protective effects of quercetin pre-treatment prior to oxidative stress with H_2_O_2_ or tBH. N = 9. Results were analyzed by paired 2-way ANOVA. *p < 0.05, **p < 0.01, ***p < 0.001, ****p < 0.0001, ns = not significant. There was no significant interaction between polarity and treatment effects for PTN, but p-values for post-hoc comparisons are still shown.

iPSC-RPE cells in 9 replicate wells were treated with H_2_O_2_ or tBH and conditioned media was collected 48 h later and analyzed by ELISA for VEGF, PEDF, CFH, and PTN (Fig. 4). Comparison of pre-treatment levels of apical and basolateral protein confirm the results from mass spectrometry. The non-significant trends of more basolateral secretion of VEGF and more apical secretion of CFH and PTN seen in mass spectrometry were confirmed and were significant using 9 replicate wells in ELISA (p < 0.0001 for VEGF and CFH, and p < 0.01 for PTN). PEDF was secreted significantly more apically, similar to the results from mass spectrometry (p < 0.0001).

H_2_O_2_ and tBH treatment significantly altered secretion of all 4 proteins, and in all cases the altered secretion was rescued at least in part with quercetin pre-treatment (Fig. 4). For all 4 proteins, the effects were greater with tBH than with H_2_O_2_. The effects of each can be seen in Fig. 4, with the effects of tBH described here. Treatment with tBH increased RPE secretion of VEGF in both apical and basolateral directions (2.4-fold and 3.4-fold, respectively). Pre-treatment with quercetin rescued this effect and reduced VEGF secretion below baseline. Two-way ANOVA with paired design showed significant main effects of both polarity (F(1,8) = 3409, p < 0.0001) and treatment (F(2,16) = 19,908, p < 0.0001), as well as a significant interaction, suggesting that oxidative stress impacted basolateral secretion more than apical secretion (F(2,16) = 5934, p < 0.0001). In contrast, tBH treatment dramatically *decreased* RPE secretion of PEDF in both apical and basolateral directions (24.0-fold and 6.9-fold, respectively). Pre-treatment with Quercetin partially rescued this decrease. Two-way ANOVA again showed significant main effects of both polarity (F(1,8) = 411, p < 0.0001) and treatment (F(2,16) = 435, p < 0.0001), as well as a significant interaction (F(2,16) = 243, p < 0.0001), demonstrating that oxidative stress impacted apical more than basolateral secretion of PEDF.

**Figure 5:**
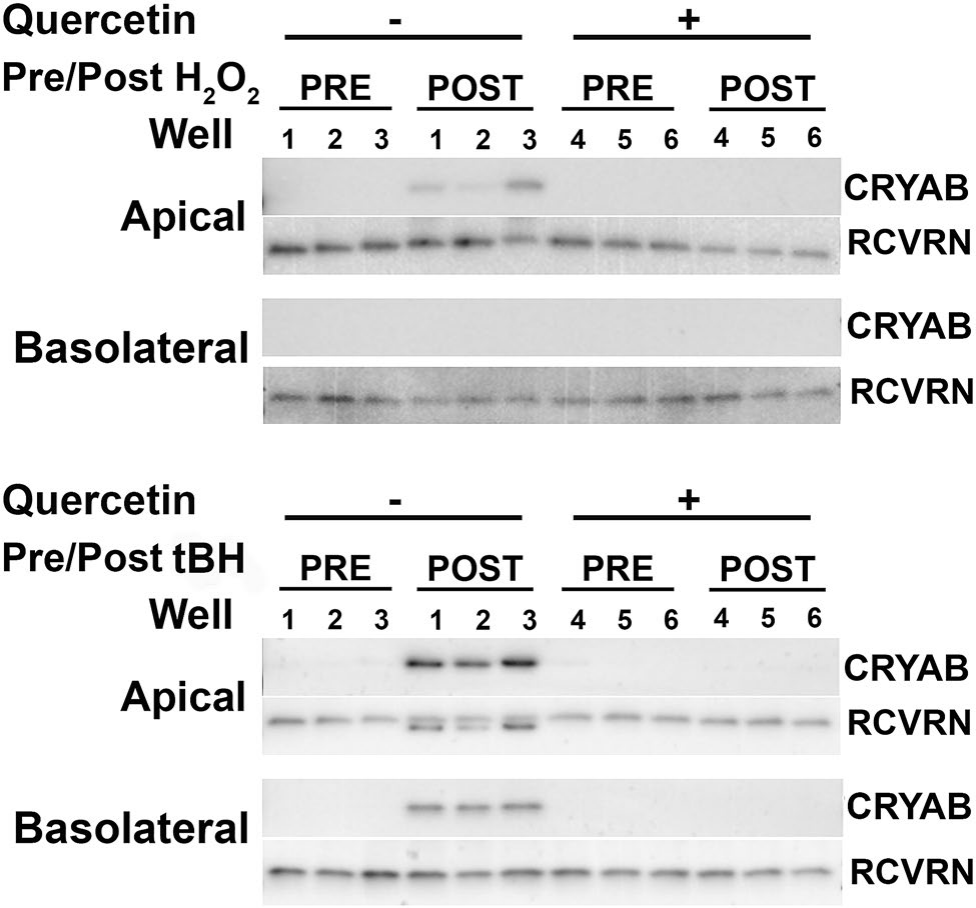
H_2_O_2_- and tBH-induced oxidative stress increase RPE polarized CRYAB secretion. Western blot shows CRYAB in apical and basolateral conditioned media before (PRE) and after (POST) incubation with (**A**) 800 µM H_2_O_2_ for 24 h, or (**B**) 1 mM tBH for 6 h, with or without pre-treatment with quercetin. As there is no endogenous protein suitable for normalization between apical and basolateral samples, equal amounts of recoverin (RCVRN) were spiked into samples and used as controls. N = 3. Cropped blots are shown. Uncropped images are in Supplementary Fig. 4.

Oxidative stress with tBH also decreased CFH secretion both apically and basolaterally (23.4-fold and 22.4-fold, respectively). While quercetin significantly rescued this effect, the rescue was smaller for CFH than the other tested proteins. There was a significant effect of polarity (F(1,8) = 135, p < 0.0001) and treatment (F(2,16) = 1820, p < 0.0001), as well as a significant interaction (F(2,16) = 95, p < 0.0001). The effects of tBH were smaller for secretion of PTN, but tBH significantly increased both apical and basolateral secretion (1.5-fold and 1.6-fold, respectively), which was rescued by quercetin. Although there was a significant main effect of polarity (F(1,8) = 8, p < 0.05) and treatment (F(2,16) = 210, p < 0.0001), there was no significant interaction between polarity and treatment, suggesting that oxidative stress impacts both apical and basolateral secretion of PTN similarly. CRYAB, assayed by western blot, was undetectable at baseline and demonstrated increased apical, but not basolateral, secretion after oxidative stress with H_2_O_2_, (Fig. 5). In contrast, tBH increased both apical and basolateral secretion of CRYAB. In all cases, ROS scavenging with quercetin prevented increased CRYAB secretion.

## Discussion

In this study, human iPSC-RPE cells were used to demonstrate directional secretion of numerous effector proteins with established roles in retinal disease. An oxidative stress model of AMD significantly altered directional secretion of proteins associated with AMD and angiogenesis in favor of a pro-angiogenic microenvironment. For VEGF, PEDF, and CFH, 2-Way ANOVA testing demonstrated a significant interaction of polarity and H_2_O_2_, suggesting that oxidative stress impacts apical and basolateral secretion differently. For CRYAB, pre-treatment quantification on ELISA was not possible as the protein was not detectable, however western blot demonstrated that oxidative stress greatly increased apical secretion and not basolateral secretion using H_2_O_2_, and both apical and basolateral secretion using tBH. This is likely due to the increased effectiveness of ROS production using tBH, as shown in Fig. 3. Altered secretion of proteins was rescued, at least in part, by ROS scavenging using quercetin. Altered directional protein secretion of RPE cells may play an important role in AMD pathophysiology.

Human iPSC-RPE was chosen to more closely model human disease and avoid the shortcomings of the commonly used ARPE19 cell line, including abnormalities in polarity and polarized secretion^28,29^. The growing field of RPE disease modeling is increasingly using iPSC-RPE cells, which can be readily generated in the lab, are terminally differentiated and non-immortalized, and do not require recurrent donations of tissue for primary culture. The iPSC-RPE line used in this study demonstrated hallmarks of bona fide RPE, including correct pigmentation, morphology, barrier properties, and RPE marker expression. Another strength of our study was the use of permeable supports to separately analyze apical and basolateral secretion. Prior studies using RPE cells grown on plastic have exposed only the apical surface to the media and therefore failed to capture changes in basolateral protein secretion. Several growth factors and cytokines in our study were secreted primarily basolateral, a finding that would be missed without the use of permeable supports. Choroidal neovascularization as well as choroidal atrophy play prominent roles in AMD pathology. Therefore, changes in basolateral growth factor and cytokine secretion may be key in pathogenesis. Our study is limited in our use of oxidative stress, which is just one proposed model of AMD among many. RPE oxidative stress is a well-established component of AMD but there are many other contributing factors.

In our model, oxidative stress increased the secretion of pro-angiogenic factors (VEGF, PTN, and CRYAB) and decreased the secretion of anti-angiogenic factors (PEDF, CFH), contributing overall to a more angiogenic environment. CFH also has a well-established role in negative regulation of complement activation and inflammation, with importance for both exudative and non-exudative AMD^18–20^. For VEGF the increased secretion effect was more basolateral than apical, which may be particularly relevant for choroidal neovascularization. Our data concur with previous studies demonstrating increased VEGF in response to oxidative stress^30,31^. H_2_O_2_ treatment of ARPE-19 cells was previously shown to reduce CFH expression, although protein secretion was not investigated^20^. Prior studies of CRYAB have demonstrated a neuroprotective effect as well as increased RNA expression in response to oxidative stress^32^. The dramatic increased apical secretion of CRYAB after oxidative stress in our RPE cells may be a compensatory mechanism for protection of the neurosensory retina. However, CRYAB has also been shown to play an important role in neovascularization, as alpha crystallin b knockout mice are resistant to retinal neovascularization in ischemic and laser-induced models^33^. Elevated CRYAB secretion may therefore contribute to exudative AMD, especially after RPE damage and breakdown of the RPE barrier.

Other studies have studied different aspects of the RPE polarized secretome. Some studies have used mass spectrometry to characterize the apical secretome only of RPE cultures in traditional culture plates and have found significant alterations in secreted proteins involved in angiogenesis and complement regulation in response to oxidative stress^7,9^. Another study investigated a subset of the RPE polarized secretome, investigating specifically proteins contained in exosomes from porcine RPE^34^. Exosomes are vesicles sized 30–150 nm carrying varied cargo including proteins, lipid, RNA, DNA, and metabolites. Four of the differentially secreted RPE effector proteins found in our study (alpha-crystallin b chain (CRYAB), annexin A1 (ANXA1), annexin A2 (ANXA2), and CD81) were also reported to be enriched in apical exosomes, consistent with the results in our study showing directional apical secretion of these proteins. Other studies have investigated the impact of different interventions, like zinc supplementation or inactivation of protease HtrA1, on the apical and basolateral RPE secretome^10,11^. These prior studies found more proteins secreted apically than basolaterally, similar to our study, although they did not specifically compare apical to basolateral secretion for any individual protein^11,34^. Similar to our study, these prior studies also used permeable supports to separate the apical and basolateral chambers. We used Transwell polyester (PET) membranes, which are 10 µM thick with 0.4 µM pores at a density of 4 × 10^6^ pores/cm^2^. We estimate that there would be approximately 3-7 pores per RPE cell. In vivo, RPE cells are separated from the choroidal vasculature by Bruch’s membrane, which is 2–5 microns thick depending on age^35^. We cannot exclude the possibility that the permeable support impedes secretion of proteins into the basolateral media and artefactually reduces protein in basolateral conditioned media, and therefore may not fully model in vivo basolateral secretion to the choroid.

In conclusion, RPE cells are highly polarized, and directional secretion of effector proteins is important for homeostasis of the adjacent neurosensory retina and choroid. Oxidative stress shifted the balance of RPE-secreted factors to favor increased angiogenesis and increased complement activation, and differentially impacted apical and basolateral secretion, which may have important implications for choroidal neovascularization. These results support future studies investigating the role of differentially secreted RPE effector proteins in photoreceptor and choroidal health to identify therapeutic targets in AMD and other retinal degenerative diseases.

## Methods

### Cell culture

iPSCs derived from healthy donor skin fibroblasts were obtained from LAgen Labs (Line 006-BIOTR-0001 Clone 1). This cell line has been used previously for iPSC-RPE differentiation and was validated at the Mayo Clinic Center for Regenerative Medicine Biotrust by expression of stem cell markers, normal karyotype, and differentiation into all 3 germ layers^36^. Our study was approved by the University of Michigan Human Pluripotent Stem Cell Research Oversight (HPSCRO) Committee and experiments were performed in accordance with their guidelines. Quality control at LAgen Labs included iPSCs staining positive for the pluripotency markers Oct4, SSEA, Nanog, and TRA-1-60, and having normal karyotyping. iPSCs were cultured on 6-well plates in mTESR1 media and passaged when reaching 50–75% confluency. iPSCs were differentiated into RPE as previously described^37^. Between Day 50 and Day 90, colonies of RPE, recognized by pigmentation and cobblestone morphology, were dissected from surrounding cells using a 21-gauge needle and passaged onto a 24-well plate coated with laminin at a density of 50,000 cells/well. After iPSC-RPE became confluent and regained appropriate pigmentation and morphology, cells were passaged onto laminin-coated Transwells. Cells were washed with phosphate buffered saline (PBS) and dissociated with trypsin to single cells and plated at a density of 335,000 cells per cm^2^. Fetal bovine serum (FBS) was added to the media at 10% for 1 week, 3% for week, and 2% for 1 week. TEER was measured once weekly starting at 4 weeks post-passage, using an EVOM device with an STX2 electrode (World Precision Instruments). For oxidative stress experiments, iPSC-RPE cells were incubated with 800 µm H_2_O_2_ for 24 h, or 1 mM tert-butyl hydroperoxide (tBH) for 6 h, then changed to maintenance media, and conditioned media was collected 48 h later.

### Detection of intracellular reactive oxygen species (ROS)

iPSC derived RPE cells were seeded in 96-well plates at 120,000/cm^2^ and maintained for 4–5 weeks before the assay. For experimental groups with quercetin treatment, cells were pre-treated with 400 µM quercetin (#10005169 Cayman) diluted in RDM medium at 37 °C for 2 h, and then rinsed twice with Hanks balanced salt solution (HBSS), prior to ROS detection. For ROS detection, cells were loaded with 10 µM Carboxy-H_2_DCFDA (6-carboxy-2′,7′-dichlorodihydrofluorescein diacetate, #C400 Invitrogen) diluted in HBSS and incubated at 37 °C for 45 min. The dye was removed, and cells were rinsed once with HBSS. 1 mM tBH (tert-Butyl hydroperoxide, #458139 Sigma) or 800 µM H_2_O_2_ was then added and incubated for 6 h and 24 h, respectively. The intracellular ROS level was measured and quantified at Ex = 485 nm and Em = 520 nm using a plate reader (Omega, BMG LABTECH). Cells treated with Carboxy-H_2_DCFDA only were used as baseline control, cells treated with tBH or H_2_O_2_ alone were used as an oxidant control, and untreated cells were used as a background control (subtracted from all other groups).

### Media collection and mass spectrometry

iPSC media was changed to RDM-B18 media, in which the B27 supplement is replaced with B18 supplement (Neurobasal medium with 0.0125% catalase, 0.625% glutathione, 0.0313% human insulin, 375 kU/L bovine superoxide dismutase, 0.025% human holo-transferrin, 192 µM T3, 7.75 mM l-carnitine, 12.5% ethanolamine, 694 mM d+-galactose, 70.9 mM putrescine, 7.23 µM sodium selenite, 1.80 mM corticosterone, 446 µM linoleic acid, 2.24 mM linolenic acid, 3.98 mM progesterone, 3.81 mM retinol acetate, 14.5 mM dl-alpha tocopherol, 26.4 mM dl-alpha tocopherol acetate, 4.43 mM oleic acid, 968 µM pipecolic acid, 512 µM biotin). Media was collected after 16 h, protease inhibitors were added, and media was immediately frozen at − 80 °C. Media was replaced with RDM maintenance media and B18 media collection was repeated once weekly. Frozen media was grouped into 3 biologic triplicates of 3 different sets of wells for the first data set, and 4 biologic replicates of 4 different sets of wells for the second data set, with age-matching between groups for number of weeks post-passage. Media was thawed and concentrated using Amicon Ultra 15 mL centrifugal filters with a 3 kDa molecular weight cutoff, with buffer exchange to phosphate buffered saline (PBS). Final protein concentration was measured using the RC DC Protein Assay (Bio Rad). When planning comparative proteomics between these 2 samples, we considered that the content and number of proteins may be disparate between apical and basolateral samples, and therefore an exogenous recombinant protein was spiked in at equal concentration in all samples to be used for normalization. Recoverin was chosen because it has several unique peptides for identification in mass spectrometry and is not expressed in RPE cells. Recombinant recoverin was added at a final concentration of 0.2 µg/µL to each sample. Final peptide abundances were normalized to recoverin and corrected for concentration factors in order to compare the total amount of each protein present in apical vs basolateral samples.

Protein samples were submitted to the University of Michigan Proteomics Resource Facility. Tandem mass tag (TMT) labeling and LC–MS/MS analysis was performed as previously described, using TMT-6 plex or TMT-16 plex (ThermoFisher) and an Orbitrap Tribid Fusion mass spectrometer (Thermo Scientific)^38^. The data were analyzed using Proteome Discoverer 2.1 (Thermo Fisher). Signal to noise values for each reporter ion in the MS/MS data were extracted and summed for each peptide and normalized based on recoverin S/N. The total intensity across channels was scaled to 100%. MS/MS spectra were searched against the UniProt human protein database^15^. Pathway analysis was performed in iPathwayGuide (Advaita Bioinformatics, Ann Arbor, MI)^12^.

### RT-PCR

Polarized iPSC-RPE cells on Transwells were harvested and RNA was extracted using the RNeasy Mini Kit(Qiagen). Complementary DNA was made with 250 ng total RNA using SuperScript II reverse transcriptase (Invitrogen), and multiplexed RT-coupled PCR was run the same day in triplicate using POWER SYBR™ Green PCR Master Mix (Applied Biosystems) for the following genes: *BEST1, CRALBP1, LRAT, MERTK, PEDF,* and *RPE65*. PCR reactions were run in the Biorad iCycler. Relative transcript levels were normalized to *ACTB.*

### Western blot

To prepare conditioned media, RDM conditioned media was collected from the apical and basolateral chambers of polarized iPSC-RPE, sodium dodecyl sulfate (SDS) buffer was added, recombinant recoverin was added for normalization at a final concentration of 0.5 nM, and media was used immediately for gel electrophoresis, enzyme-linked immunoassay (ELISA), or stored at − 80 °C. Proteins were separated via SDS–polyacrylamide electrophoresis (SDS/PAGE) and transferred onto PVDF membranes. The membranes were blocked with tris buffered saline (TBS) containing 2% fetal bovine serum (FBS) and 0.5% tween-20, incubated with primary antibody overnight, washed, and incubated with horse radish peroxidase (HRP)-conjugated secondary antibody for 1 h. Blots were developed with EcoBright Femto HRP (Innovative Solutions). Bands were visualized and photographed using an Azure c500 (Azure Biosystems). Densitometry quantification was performed using ImageJ software. The primary antibodies used were: anti-CRYAB (Enzo, ADI-SPA-223), anti-RCVRN (Millipore, AB5585), and anti-RCVRN (Proteintech, 66521-Ig).

### Enzyme-linked immunosorbent assay (ELISA)

The ELISA kits for VEGF (R&D Systems, SVE00), PEDF (R&D Systems, DY117705), CFH (R&D Systems, DY4779), and PTN (Invitrogen, EH370RB) were used per the manufacturer’s instructions. In the case of the PEDF and CFH ELISA kits, the wells were coated with capture antibody (diluted in PBS) overnight at room temperature. The wells were then blocked with Reagent Diluent for 1 h at room temperature. In the case of the VEGF and PTN ELISA kits, the plates came pre-coated and pre-blocked. The conditioned media was diluted with assay diluent, added to the pre-coated plate in triplicate, and incubated for 2 h at room temperature. After washing, anti-VEGF, anti-PEDF, anti-CFH, or anti-PTN conjugated with HRP was added to the plate and incubated for 2 h at room temperature. After developing with H_2_O_2_ and the chromogen tetramethylbenzidine, the optical density of each well was determined using a microplate reader. Standard concentrations of recombinant VEGF, PEDF, CFH, or PTN were used in parallel to generate a standard curve to calculate concentrations.

### Immunocytochemistry

Cells were washed in phosphate buffered saline and fixed with 4% paraformaldehyde for 15 min, washed and blocked for 1 h in blocking buffer (3% bovine serum albumin (BSA), 0.3 M glycine, 0.15% Triton X-100, 1% donkey serum). Cells were incubated in primary antibody overnight at 4 °C, washed, and incubated in secondary antibody with DAPI (ThermoFisher) and phalloidin conjugate (Biotium, 00050-T). Primary antibodies used were: anti-ZO-1 (Invitrogen, 33-9100), anti-CD147 (BDPharmigen, 563020), anti-ezrin (Invitrogen, MA5-13862), and anti-kir7.1 (Santa Cruz, sc-22438). Secondary antibodies used were: goat anti-mouse IgG (Jackson Immunoresearch, 715-545-150) and donkey anti-rabbit IgG (Jackson Immunoresearch, 711-545-152).

### Statistical analysis

For proteomics results, P-values were calculated using the Proteome Discoverer software using a student T test to compare the apical and basolateral means of each protein. Given the large number of proteins being compared, adjustment for multiple comparisons employed the Benjamin and Hochberg false discovery rate approach. At least a 2.0-fold change and an adjusted p-value of < 0.05 was considered significant. For analysis of molecular functions of differentially secreted proteins in Fig. 2B, analysis was performed by Advaita iPathwayGuide as previously described^13^. Briefly, the program calculated the probability of observing a number of differentially secreted proteins annotated to a given molecular function that is equal to or greater than the actual observed number of differentially secreted proteins for that molecular function, and the p-value was corrected for false discovery rate. For ROS detection studies, an unpaired 1-way ANOVA was used with a Tukey correction for multiple comparisons for the tBH experiments, and a Kruskal–Wallis test with a Dunn’s correction was used for the H_2_O_2_ experiments due to unequal sample size number resulting in unequal variance. For ELISA results before and after oxidative stress, a paired 2-way ANOVA was used with a Tukey correction for multiple comparisons. P-values < 0.05 were considered statistically significant.

## Supplementary Information

Supplementary Figures.
