# Supplementary material for: Oxidative stress differentially impacts apical and basolateral secretion of angiogenic factors from human iPSC-derived retinal pigment epithelium cells
Source: Sci Rep. 2022 Jul 26;12:12694. doi: 10.1038/s41598-022-16701-6 (PMC9325713; doi:10.1038/s41598-022-16701-6)
Supplement: Supplementary file 1 — Supplementary Figures. [file 41598_2022_16701_MOESM1_ESM.pdf]

**SUPPLEMENTARY MATERIAL**

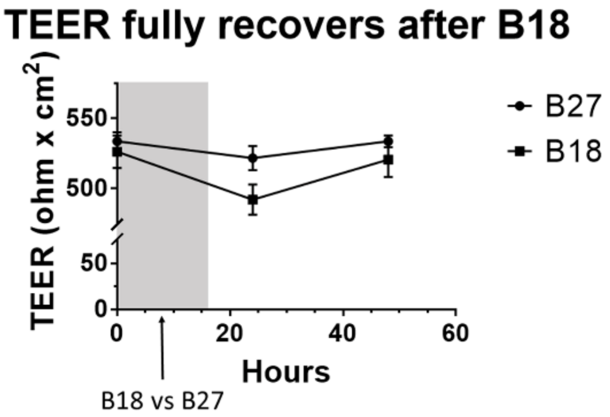


**Supplementary Figure 1**. **Assessing RPE function after incubation with albumin-free B18 media.** Graph shows change in TEER over time in iPSC-RPE supplemented with either B27 or B18 for 16 hours, after which both cultures were changed to fresh B27-supplemented media. Paired 2-way ANOVA using a Geisser-Greenhouse correction showed significant effects of time ($F(1.0,4.1)=235$, $p<0.0001$) and a significant interaction between time and media type ($F(2,8)=49.5$, $p<0.0001$), but no significant effect of media ($F(1,4)=4.72$, $p=0.10$). There was no difference between B18 and B27 at any time point in post-hoc comparisons. N=3, data points show mean $\pm$ SD.

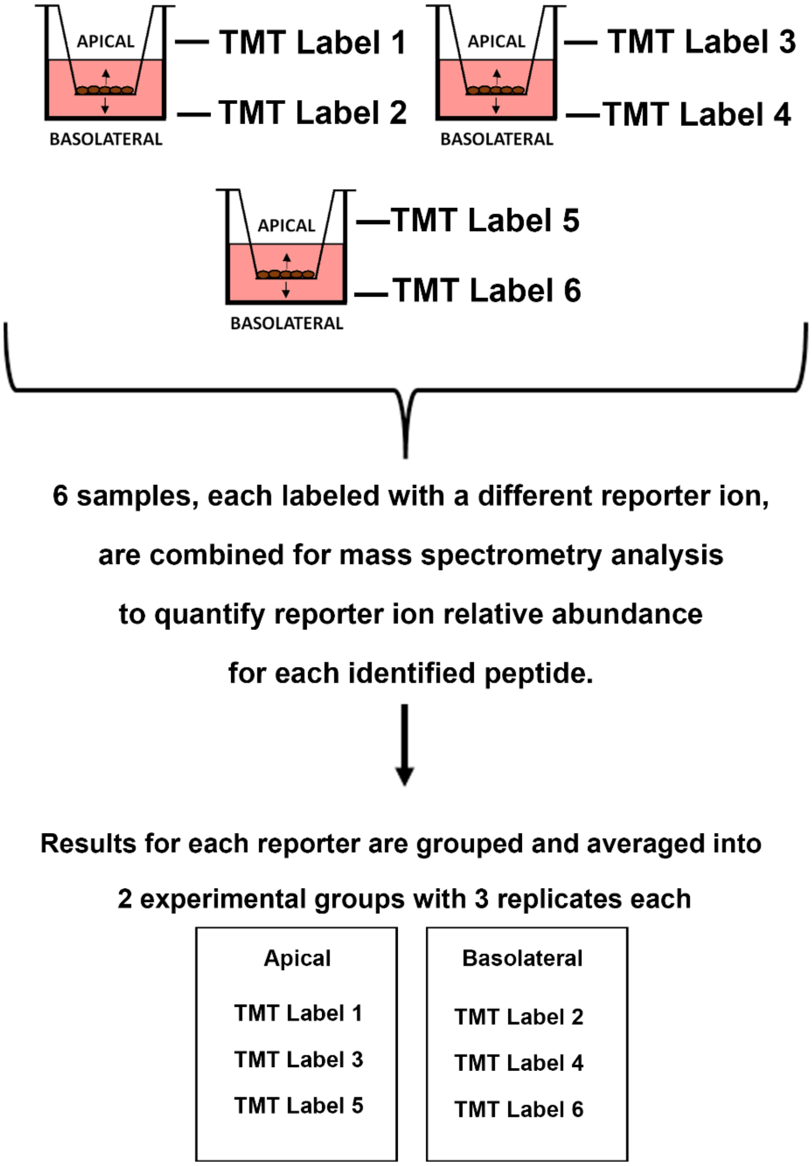


**Supplementary Figure 2. Tandem Mass Tag Spectrometry Experimental Design.** Design is shown for TMT 6-plex, used for Data set 1 in the results section. For Data set 2, 4 replicates were used instead of 3. Although 1 well is shown per replicate for simplicity of the diagram, multiple wells were used for each replicate in order to pool conditioned media to obtain adequate protein for mass spectrometry.

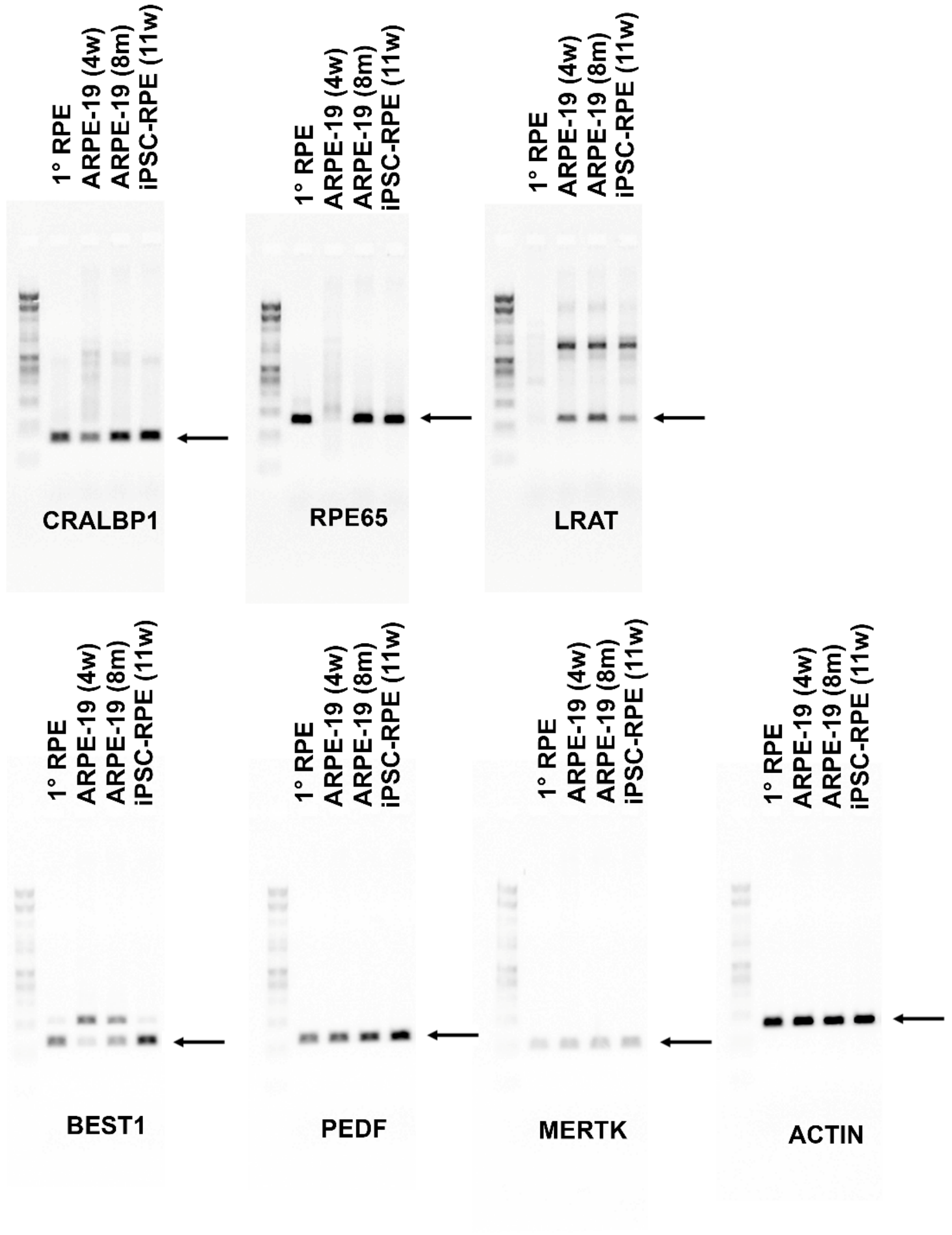


**Supplementary Figure 3. RT-PCR of RPE marker genes.** Full versions of the cropped photos shown in **Figure 1C** of RT-PCR products run on agarose gels. 1° RPE = primary adult RPE culture, ARPE-19 (4w) = ARPE-19 cells 4 weeks post-passage, ARPE-19 (8m) = ARPE-19 cells 8 months post-passage, and iPSC-RPE (11w)= iPSC-RPE cells 11 weeks post-passage. The arrows indicate the expected size of PCR product.

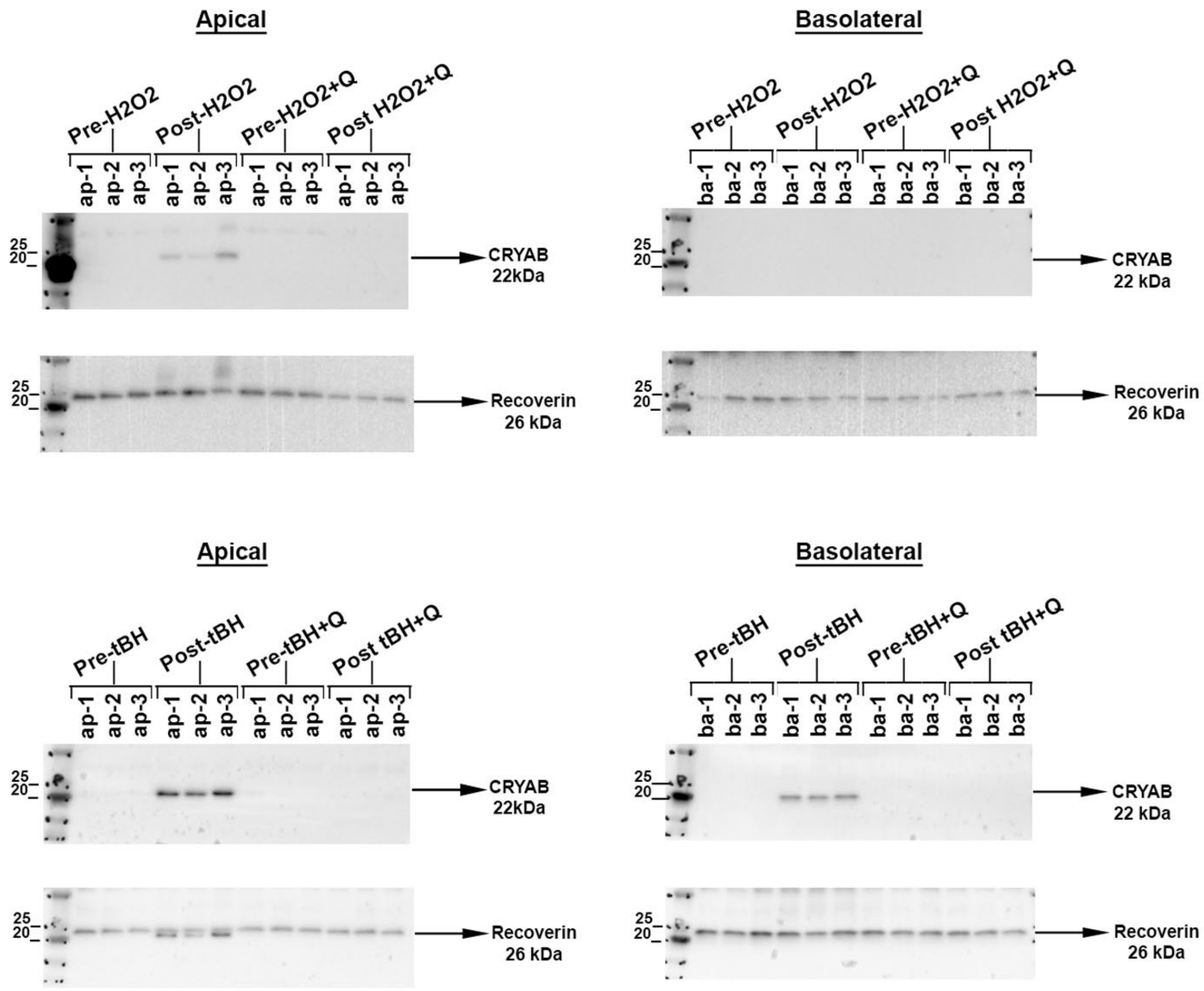


**Supplementary Figure4. Western blots of CRYAB and Recoverin.** Full-view images of Western blots used for cropped images in **Figure 5**.
